# The Insurance Acts and the Hospitals

**Published:** 1914-02-21

**Authors:** Richard Kershaw

**Affiliations:** Central London Throat and Ear Hospital, Gray's Inn Road


*To the Editor of* The Hospital.

### The Insurance Acts and the Hospitals.

*To the Editor of* The Hospital.

Sir,—It is but a little over a year since hospital authorities were naturally agitated concerning the influence which the National Insurance Act would exercise upon the medical and the financial resources of the voluntary hospitals of this country.

Many and various resolutions were passed as to the future regulations which were to be the guiding principles for the admission of insured persons to the benefits of our institutions. From time to time we heard suggestions of the most drastic measures which were to be taken; in some cases all insured persons were to be denied admission to the out-patient department; in others, all so-called trivial cases were to be excluded, while yet again only those patients were to be admitted who were sent to the hospital on the recommendation of a medical practitioner.

It would be interesting to learn to what extent any of these measures have been carried out in reality; especially interesting would it be to learn what amount of diminution has taken place in the number of out-patients during last year.

On the occasion when Mr. Worthington Evans gave his interesting and lucid address to the members of the Hospital Officers' Association at St. Bartholomew's Hospital, I ventured to prophesy that the Insurance Act would not diminish the out-patient work at the special hospitals, and, further, that it would be the means of making these institutions what many of us have long been striving for—viz., consultation centres to which the general practitioners would be attracted not only because they were centres for relief, but because they were also centres for teaching.

In this connection, then, I venture to give some figures taken from the record of this institution, and if it were possible to obtain statistics of a like nature from other hospitals, they would throw an interesting light upon the question.

For obvious reasons of comparison the figures are given for the last two years :—

| — | 1912 | | 1913 | |
|---|---|---|---|---|
| | New Out-patients | Sent by Doctors | New Out-patients | Sent by Doctors |
| Jan., Feb., March... | 2,760 | 1,160 =42 % | 2,485 | 1,198 =48.2 % |
| April, May, June ... | 3,012 | 1,254 =41 % | 2,870 | 1,525 =53.1 % |
| July, Aug., Sept. ... | 2,769 | 1,104 =39.5 % | 2,948 | 1,526 =52 % |
| Oct., Nov., Dec. ... | 2,495 | 996 =39 % | 2,548 | 1,322 =52 % |
| Total ... ... | 11,036 | 4,514 =40 % | 10,851 | 3,571 =51 % |

The in-patients numbered 977 in 1912, and 1,031 in 1913, the percentage sent by doctors being 64 per cent. and 66 per cent. respectively.

So much, then, for the medical aspect in relation to the relief of the sick poor.

As to the teaching aspect, the following figures speak for themselves, and illustrate that my prediction was not far out :—

*Medical Men Visiting the Hospital to Witness the Practice of the Staff.*

| 1911 | 1912 | 1913 |
|---|---|---|
| 636 | 672 | 1,399 |

In addition to these visitors, thirty gentlemen enrolled themselves as graduate students, taking out a course of instruction for periods varying from three to six months.

I am, yours faithfully,

Richard Kershaw.

Central London Throat and Ear Hospital,
Gray's Inn Road, February 12, 1914.

[We shall be glad to have the experience of other hospital superintendents and secretaries on some or all of the points raised in this letter.—Ed. The Hospital.]

